# HOW TO GET AN NIA GRANT

**DOI:** 10.1093/geroni/igac059.807

**Published:** 2022-12-20

**Authors:** Kenneth Santora

**Affiliations:** National Institute on Aging, Bethesda, Maryland, United States

## Abstract

Dr. Santora will provide an overview of the NIA application process and will share information on relevant policy changes.

